# Dietary nitrate reduces skeletal muscle oxygenation response to physical exercise: a quantitative muscle functional MRI study

**DOI:** 10.14814/phy2.12089

**Published:** 2014-07-22

**Authors:** Rachel Bentley, Stuart R. Gray, Christian Schwarzbauer, Dana Dawson, Michael Frenneaux, Jiabao He

**Affiliations:** 1 Aberdeen Biomedical Imaging Centre, School of Medicine and Dentistry, University of Aberdeen, Aberdeen, U.K; 2 Musculoskeletal Research Programme, School of Medicine and Dentistry, University of Aberdeen, Aberdeen, U.K; 3 Cardiovascular Research Programme, School of Medicine and Dentistry, University of Aberdeen, Aberdeen, U.K

**Keywords:** Magnetic resonance imaging, mitochondria, nitrate, peripheral vascular function, skeletal muscle exercise

## Abstract

Dietary inorganic nitrate supplementation (probably via conversion to nitrite) increases skeletal muscle metabolic efficiency. In addition, it may also cause hypoxia‐dependent vasodilation and this has the potential to augment oxygen delivery to exercising skeletal muscle. However, direct evidence for the latter with spatial localization to exercising muscle groups does not exist. We employed quantitative functional MRI (fMRI) to characterize skeletal muscle oxygen utilization and replenishment by assessment of tissue oxygenation maximal change and recovery change, respectively. Eleven healthy subjects were enrolled, of whom 9 (age 33.3 ± 4.4 years, five males) completed the study. Each subject took part in three MRI visits, with dietary nitrate (7cl concentrated beetroot juice) consumed before the third visit. During each visit fMRIs were conducted concurrently with plantar flexion exercise at workloads of 15% and 25% maximum voluntary contraction (MVC). No significant changes were found between visits 1 and 2 in the fMRI measures. A decrease in maximal change was found at 15% MVC in soleus between visits 2 and 3 (5.12 ± 2.36 to 2.55 ± 1.42, *P* = 0.004) and between visits 1 and 3 (4.43 ± 2.12 to 2.55 ± 1.42, *P* = 0.043), but not at 25% MVC or within gastrocnemius. There was no difference in recovery change between visits. We found that dietary nitrate supplementation reduces tissue oxygenation alterations during physical exercise in skeletal muscle. This effect is more prominent in muscles with predominantly type 1 fibers and at lower workloads. This indicates that in healthy subjects dietary nitrate predominantly affects skeletal muscle energy efficiency with no change in oxygen delivery.

## Abstract (precis)

e12089

It is known that nitrate supplement improves skeletal muscle metabolic efficiency, and postulated to be attributed to improved oxygen delivery to the tissue. We examines the effects of dietary nitrate on skeletal muscle oxygen regulation in healthy subjects under two levels of physical exercise intensities, and found that the tissue oxygenation change induced by physical exercise is reduced by nitrate supplement primarily at a lower exercise intensity in type 1 muscle fibers. Our results indicate that the hypoxic stress associated with a higher exercise intensity does not augment the oxygen delivery to skeletal muscle in healthy subjects.

## Introduction

Skeletal muscle function is often altered by ischemic vascular diseases, such as peripheral arterial occlusive disease (PAOD) (Casaburi 2001; Criqui MaN 2006). Dietary inorganic nitrate (found in high concentrations in green leafy vegetables and in beetroot) supplementation has been shown to improve skeletal muscle metabolic efficiency during exercise and in hypoxic environments (Engan et al. 2012), and has blood pressure lowering and antiplatelet effects (Webb et al. 2008). Dietary nitrate is absorbed from the gut, taken up into the salivary glands and concentrated in the saliva (Kapil et al. 2013). It is reduced by salivary bacteria to nitrite which is absorbed into the bloodstream (Webb et al. 2008). Nitrite rather than nitrate appears to be responsible for the aforementioned actions, as use of mouthwash prevents the rise in plasma nitrite following a single dose of oral nitrate and prevents the blood pressure lowering and antiplatelet effects (Kapil et al. 2013). It has also been shown that nitrite infusion into the human forearm increases forearm blood flow at rest and during exercise in normoxia (Cosby et al. 2003). Nitrite is converted under hypoxic and/or acidotic conditions to nitric oxide and this appears to be responsible for at least some of the bioactivity of nitrite, although nitrite may also exert direct effects via post‐translational S‐nitrosylation of proteins (Thomas and Jourd'heuil 2012).

Given the previously reported hypoxic augmentation of nitrite‐induced vasodilation (Maher et al. 2008), it is possible that nitrate supplementation may also enhance oxygen delivery to exercising skeletal muscle as well as increasing the efficiency of oxygen utilization. Such an effect would be of particular importance in patients with peripheral arterial disease (Kenjale et al. 2011). A previous study investigating oxygen delivery to exercising skeletal muscle using near infra‐red spectroscopy (NIRS) (Bailey et al. 2009), found fractional oxygen extraction was reduced after nitrate supplementation. However, NIRS measures are spatially localized to superficial tissues. Therefore, it remains unclear whether nitrate supplementation increases oxygen delivery to exercising skeletal muscle as well as the efficiency of energy generation. We therefore hypothesized that an acute single dose of dietary nitrate would reduce tissue oxygenation alterations to a physical exercise challenge with a greater reduction in this alteration at a higher exercise intensity.

To probe this hypothesis, we conducted quantitative muscle functional magnetic resonance imaging (fMRI) (Boushel et al. 2000; Meye and Prior 2000; Akima et al. 2004; Damon and Gore 2005; Kinugasa et al. 2006; Damon et al. 2007a) for spatially resolved assessment of tissue oxygenation during plantar flexion exercise, with the soleus and gastrocnemius as the well‐defined activated muscle groups (Kinugasa and Akima 2005). The utilization and recovery of tissue oxygenation can be characterized by maximal change and recovery change of the fMRI time course. Two exercise workloads were adopted to examine the differences in muscle responses at varying intensities, and in turn the energy expenditure and hypoxic stress.

We investigated whether there is an alteration in the amount of oxygenation change induced by varying exercise intensities and its recovery as the result of dietary nitrate, and if such alteration is related to systemic physiological conditions.

## Methods

**Figure 1.**
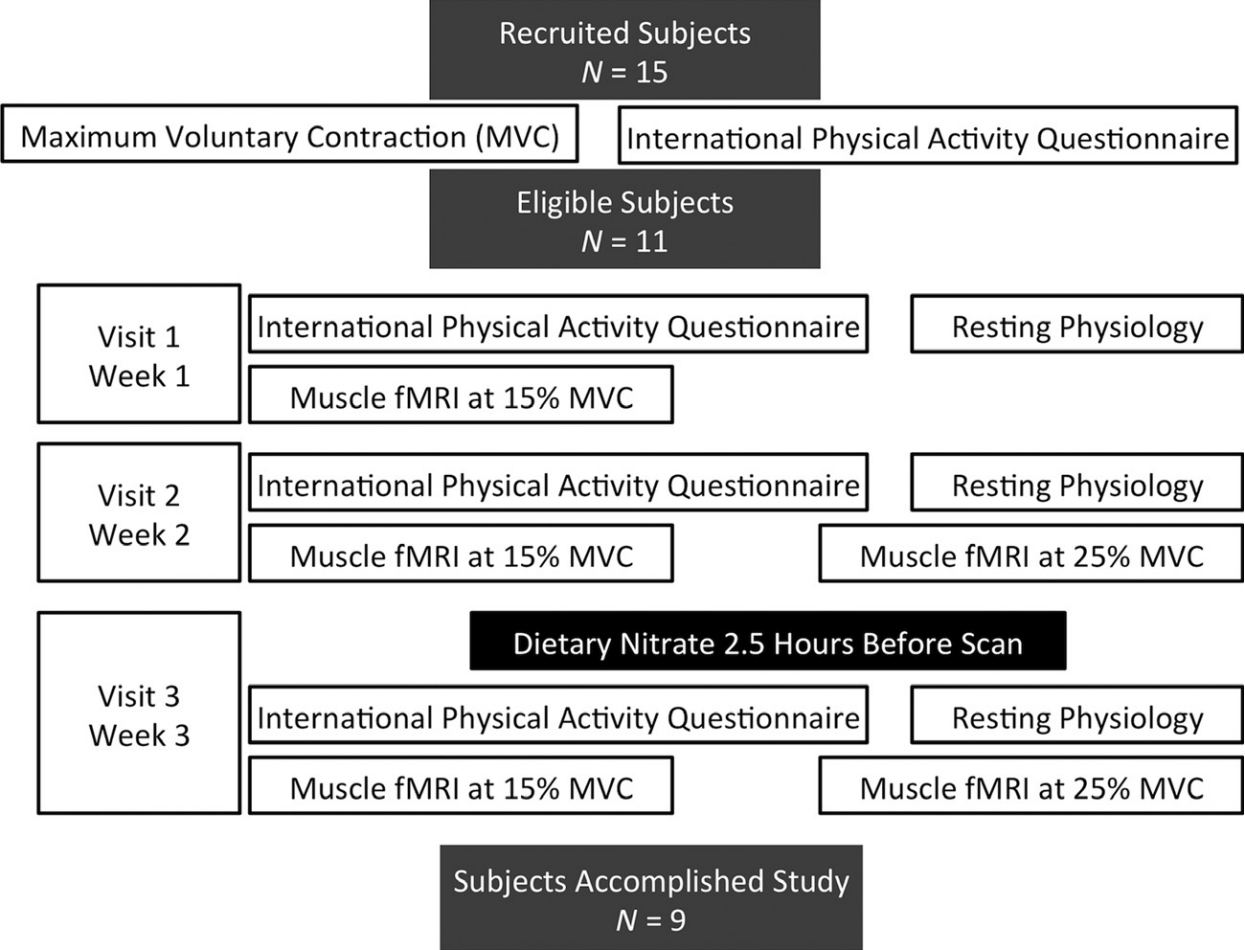
Study design. Among 15 recruited subjects, 11 met the inclusion criteria and nine completed the study. Eligible subjects were invited to take part in three MRI visits with a week between consecutive visits, where each visit consisted of resting physiological recording, immediately followed by two functional MRI runs at 15% and 25% of maximum voluntary contraction (MVC). International physical activity questionnaire (IPAQ) was collected at each visit to monitor variations in exercise pattern. Subjects were instructed to consume dietary nitrate in the form of 7cl concentrated beetroot juice 2.5 h before the third visit.

After screening, each subject took part in three separate MRI visits, with at least 1 week between consecutive visits. Visits 1 and 2 were conducted under identical control conditions in order to test the reproducibility of the measurement, while visit 3 was conducted 2.5 h after the consumption of 7cl (0.4 g/2.58 mmol nitrate per dose) concentrated beetroot juice (SPORT shot, James White Drinks, Suffolk, U.K.). The participants were instructed not to use antibacterial mouthwash on the day of scan visits. Each MRI visit involved resting physiological monitoring followed by two fMRI runs (Fig. 1). Subjects were instructed to refrain from consumption of alcohol or caffeine and to avoid any strenuous exercise within the 24 h period before each visit. The study was approved by the local ethics committee (College Ethics Review Board, University of Aberdeen), and written informed consent was obtained prior to the study.

### Subjects

Among the 15 subjects who expressed interest, 11 met the inclusion criteria and 9 (age 33.3 ± 4.4 years, five males) completed the study. Subjects were excluded if they presented any cardiovascular or musculoskeletal conditions. Subjects with conditions incompatible with MRI were excluded from the study. Eligible subjects were not taking prescribed or over the counter medications during the study. Isometric maximal voluntary contraction (MVC) of the right calf with the ankle at a 90 degree angle during plantar flexion for each subject was measured (KinCom500H dynamometer; KinCom, East Ridge, TN). Measurements at 2 min intervals were performed until the difference between the last two measurements were <5% of their average, normally 3–4 repetitions were performed. The highest of the last two measurements was taken as the MVC. An International Physical Activity Questionnaire (IPAQ) was employed for estimating habitual physical activity levels undertaken during the preceding 7 days. The questionnaire was repeated before each visit for monitoring purposes. Subjects, with IPAQ > 10,000 MET‐mins or MVC outside the range of 200–700 N, were excluded.

### Physiological measurement

Physiological measurements were conducted at rest at the beginning of each visit, using a MRI compatible monitor (Schiller MAGLIFE Serenity, Wissembourg, France). Oxygen saturation, heart rate, breathing rate, inspired and end tidal CO_2_ were recorded during for the final 5 min of a 10 min supine rest period. The mean of the physiological recordings were subsequently computed as the characteristic values. Three blood pressure (BP) measurements were taken at 5 min intervals during supine rest, and the mean of the second and third measurements was computed as the BP.

### Image acquisition

**Figure 2.**
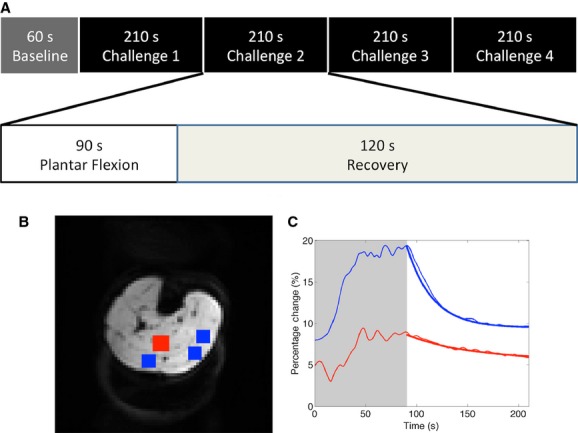
Functional imaging acquisition and analysis: (A) schematic diagram showing exercise protocol, (B) location of region of interests in a typical subject and (C) cycle averaged percentage time course of a typical functional MRI run. (A) The schematic diagram shows the exercise protocol. Each functional MRI run consisted of 60 sec resting baseline, followed by four 210 sec exercise cycles. Each cycle was composed of 90 sec plantar flexion exercise at 0.3 Hz followed by a 120 sec recovery. (B) The location of region of interests (ROIs) are shown on the functional image in a typical subject. Rectangular ROIs were defined manually for soleus (red) as well as medial and lateral gastrocnemius (blue), with fixed number of voxels across all subjects in corresponding muscle groups. (C) The cycle averaged time course in a typical functional run is shown with shaded area indicating the 90 sec plantar flexion exercise. The time course in soleus (red) and gastrocnemius (blue) are shown in thin lines, while the single exponential fitting of the recovery time course is shown by bold lines.

Imaging was performed on a 3 T MRI scanner (Achieva; Philips Healthcare, Best, The Netherlands), using a body coil for transmission and a SENSE knee coil as receiver. Each scan visit consisted of a standard clinical anatomical protocol, standard clinical phase contrast angiogram and two fMRI runs, performed on the right calf. Quantitative functional images sensitive to changes in the blood oxygenation level‐dependent (BOLD) contrast were acquired using a dual‐gradient‐echo echo‐planar imaging (EPI) sequence with a repetition time (TR) of 3 sec, echo times (TE1/TE2) of 21/60 msec and a flip angle of 90 degrees. Ten contiguous 5‐mm thick slices were acquired with field of view (FOV) was 200 × 200 mm^2^ and the matrix size of 80 × 80, which resulted in a voxel size of 2.5 × 2.5 × 5 mm^3^. The imaging volume was centered at the maximal circumference of the calf. The fMRI acquisition was performed concurrently with plantar flexion exercise, which was composed of a 60 sec baseline and four cycles of 210 sec stimulus (Fig. 2A). Each cycle was comprised of 90 sec plantar flexion exercise at 0.3 Hz with a fixed amount of weight load, and 120 sec recovery period. Two consecutive fMRI runs were conducted in each visit with fixed workloads of 15% and 25% of MVC.

### Image analysis

The acquired fMRI images were corrected for movement and spatially smoothed using a Gaussian kernel with a full‐width‐at‐half maximum (FWHM) of 3 × 3 ×6 mm^3^; these processing steps were carried out using the SPM8 software package (Frackowiak 2004). Rectangular regions of interest (ROIs) were defined in ImageJ (National Institutes of Health, Bethesda, MD) manually on the middle six slices (Fig. 2B) corresponding to medial gastrocnemius (32 × 6 voxels), lateral gastrocnemius (16 × 6 voxels) and soleus (25 × 6 voxels) (Ledermann et al. 2006). Medial and lateral gastrocnemius were combined as the gastrocnemius of 48 × 6 voxels. Reference was made to the anatomical and angiographic images to ensure the conservative definition of ROIs and the exclusion of large vessels and adipose fat. Tissue oxygenation time courses, indicated by effective transverse relaxation rate, were subsequently extracted in Matlab (MathWork, Natick, MA) by applying the ROIs to the fMRI time series and computing the relaxation rate based on Bloch equations (Ledermann et al. 2006; Damon et al. 2007a). Baseline values were computed as the mean from 15 to 30 sec of each time course, while the cycle averaged time courses were calculated as the average of the final three cycles of each time course to eliminate muscle memory effects (He et al. 2013). The cycle averaged time courses were subsequently normalized by the baseline values to derive the percentage change time courses. Single exponential fitting was then performed on the post exercise recovery portion of each percentage change time course, and the tissue oxygenation maximal change (peak amplitude) and recovery change (peak to trough normalized by peak amplitude) were extracted (Fig. 2C). The image analysis procedure was predefined before the study, and carried out by a researcher independent of the scanning procedure and statistical analysis.

### Statistical analysis

Statistical analysis was performed using SPSS statistical software (Version 21; IBM, Armonk, NY). The normality of observed parameters were assessed by the Kolmogorov–Smirnov test. One‐way ANOVA tests with repeated measures were performed on resting physiological measurements to identify any systemic physiological shift and on tissue oxygenation maximal change and recovery change for both the soleus and gastrocnemius at both 15% and 25% MVC. The changes in the maximal change and recovery change from visit 2 to 3, were then correlated with changes in BP. *P*‐value of 0.05 was adopted as the threshold of statistical significance.

## Results

Subject demographic information, MVC, resting physiological characteristics and physical activity levels are shown in Table 1.

**Table 1.** Subject demographic information, resting physiological characteristics, physical activity levels and muscle strength.

Parameters	Visit 1	Visit 2	Visit 3
Age	33.3 ± 4.4	–	–
Gender	5 male	–	–
Weight (kg)	72 ± 12.2	–	–
Height (cm)	173.5 ± 10.5	–	–
Body Mass Index	23.7 ± 2.5	–	–
Maximum voluntary contraction (N)	451 ± 143	–	–
Systolic blood pressure (mmHg)	113.44 ± 7.72	106.89 ± 4.51	106.78 ± 9.24
Diastolic blood pressure (mmHg)	70.06 ± 5.11	66.89 ± 6.74	64.94 ± 5.89
Oxygen saturation (%)	98.81 ± 0.44	97.73 ± 3.01	97.02 ± 1.18
Heart rate (beats/min)	63.70 ± 8.80	59.72 ± 12.34	60.37 ± 6.56
Breathing rate (breaths/min)	14.60 ± 3.83	14.67 ± 3.90	14.23 ± 4.04
Inspired CO_2_ (mmHg)	1.04 ± 0.92	0.54 ± 0.46	1.00 ± 1.05
End tidal CO_2_ (mmHg)	38.82 ± 3.58	38.07 ± 3.17	39.80 ± 3.99
Physical activity score (min)	3844 ± 2800	2830 ± 1191	5494 ± 4625

The mean and standard deviation of subject demographic information and maximum voluntary contraction are shown for the group, as well as the resting physiological characteristics and physical activity level for each visit. There was no significant difference in physiological characteristics between visits 2 and 3.

**Figure 3.**
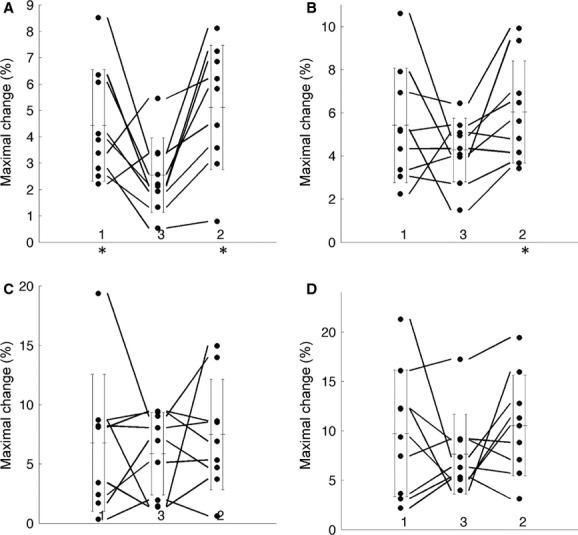
Tissue oxygenation maximal change at 15% and 25% maximum voluntary contraction for all the three visits of (A) soleus at 15% MVC, (B) gastrocnemius at 15% MVC., (C) soleus at 25% MVC and (D) gastrocnemius at 25% MVC. The tissue oxygenation maximal change of each subject is represented by a circle, with the measurement of the same visit shown in the same column. The mean and standard deviation of the visit are shown as the error bar on both sides of the corresponding column. The subjects are identified across all the visits by connection lines. No significant change was found between visits 1 and 2. Significant differences are marked by “*”. The three visits are presented in visits 1, 3, and 2 order, so that the effects of dietary nitrate (visit 3) can be more easily contrasted with visits 1 and 2.

At 15% MVC, tissue oxygenation maximal change showed no significant difference between visits 1 and 2. However, a significant decrease in tissue oxygenation maximal change was observed in soleus between visits 1 and 3 (4.43 ± 2.12 to 2.55 ± 1.42, *P* = 0.043), as well as between visits 2 and 3 (5.12 ± 2.36 to 2.55 ± 1.42, *P* = 0.004) (Fig. 3, Table 2). This decrease in gastrocnemius was not significant between visits 1 and 3 (5.43 ± 2.66 to 4.28 ± 1.47, *P* = 0.234), but significant between visits 2 and 3 (6.04 ± 2.36 to 4.28 ± 1.47, *P* = 0.018) (Fig. 3, Table 2). Tissue oxygenation recovery change did not show significant differences between visits (Table 2). At 25% MVC, no significant differences were found for either maximal change or recovery change between visits (Table 2).

**Table 2.** Comparison of tissue oxygenation between the three visits.

MVC Level	Measurement	Muscle group	Visit 1	Visit 2	Visit 3	*P*‐value 1 vs. 2	*P*‐value 2 vs. 3	*P*‐value 3 vs. 1
15%	Maximal change	Gastrocnemius	5.43 ± 2.66	6.04 ± 2.36	4.28 ± 1.47	0.559	0.018	0.234
		Soleus	4.43 ± 2.12	5.12 ± 2.36	2.55 ± 1.42	0.510	0.004	0.043
	Recovery change	Gastrocnemius	2.49 ± 3.04	2.65 ± 2.33	2.92 ± 1.87	0.863	0.599	0.581
		Soleus	0.04 ± 1.34	0.97 ± 1.69	1.21 ± 1.14	0.273	0.540	0.086
25%	Maximal change	Gastrocnemius	9.05 ± 7.48	9.84 ± 6.47	7.63 ± 4.03	0.655	0.294	0.547
		Soleus	6.78 ± 5.79	7.50 ± 4.67	5.87 ± 3.47	0.530	0.361	0.597
	Recovery change	Gastrocnemius	4.60 ± 3.68	4.39 ± 3.65	5.32 ± 3.25	0.773	0.287	0.491
		Soleus	0.66 ± 1.87	0.80 ± 1.41	0.81 ± 1.35	0.871	0.988	0.750

The mean and standard deviation of tissue oxygenation maximal change and recovery change are shown for each muscle group, exercise stress level, and scan visit. The statistical result of paired *t*‐test between visits are shown, with *P* < 0.05 highlighted. The visits 1 and 2 were under identical conditions, while visit 3 was after dietary nitrate supplementation.

A significant correlation between the change in systolic BP and tissue oxygenation maximal change in gastrocnemius at 25% MVC between visits 1 and 3 was observed (*r* = −0.7810, *P* = 0.0130, Table 3). There were no other significant correlations between the changes in tissue oxygenation measures between visits and corresponding changes in BP (Table 3).

**Table 3.** Correlation between changes in tissue oxygenation measures and changes in blood pressure (BP) between visits.

MVC level	Measurement	Muscle group	Changes in systolic BP			Changes in diastolic BP		
			1–2 *P*‐value	2–3 *P*‐value	3–1 *P*‐value	1–2 *P*‐value	2–3 *P*‐value	3–1 *P*‐value
15%	Maximal change	Gastrocnemius	0.8400	0.2779	0.9626	0.6155	0.8377	0.6012
		Soleus	0.4671	0.5743	0.5745	0.8134	0.4474	0.9937
	Recovery change	Gastrocnemius	0.4130	0.7467	0.9116	0.8427	0.7020	0.9089
		Soleus	0.7690	0.6485	0.4693	0.2738	0.8645	0.3044
25%	Maximal change	Gastrocnemius	0.0715	0.9817	0.0130	0.2835	0.8884	0.6722
		Soleus	0.8082	0.8169	0.2809	0.3860	0.4699	0.4388
	Recovery change	Gastrocnemius	0.5792	0.0642	0.8862	0.6660	0.5093	0.7580
		Soleus	0.7993	0.5199	0.7331	0.5263	0.8099	0.5819

The changes in systolic and diastolic BP were correlated with the change in tissue oxygenation measures in each muscle group and exercise intensity. The *P*‐value is given for each correlation with *P* < 0.05 highlighted. The visits 1 and 2 were conducted in identical conditions, while visit 3 was performed after dietary nitrate supplementation.

## Discussion

In this study, we have employed a quantitative fMRI method to examine the effects of dietary nitrate supplementation on tissue oxygenation in skeletal muscle during physical exercise. We found that the tissue oxygenation maximal change was reduced by dietary nitrate at a workload of 15% of MVC in soleus, but not gastrocnemius, muscles. However, when exercising at 25% MVC this reduction was not statistically significant. We did not observe any significant alterations in oxygenation recovery at either workload with nitrate supplementation. The significant reduction in tissue oxygenation maximal change in soleus at 15% MVC were not correlated with BP.

Our results agree with previous findings that dietary nitrate alters skeletal muscle energetics and oxygen regulation (Bailey et al. 2009, 2010; Larsen et al. 2011, 2014). Previous preclinical studies have indicated that increased levels of endogenous nitric oxide cause decreases in oxygen consumption and improves the associated coupling with ATP turnover (Shen et al. 1994, 2001), suggesting an enhanced efficiency of energy production. In humans, supplementation with nitrate has been shown to reduce the oxygen cost of exercise (Bailey et al. 2009, 2010). In another study, dietary nitrate supplementation reduced the expression of the mitochondrial protein Adenine Nucleotide translocase (ANT) which uncouples the electron transport chain (Larsen et al. 2011), suggesting enhanced mitochondrial energy coupling. Furthermore, in isolated skeletal muscle mitochondria from these subjects, mitochondrial uncoupling was reduced under hypoxic but not normoxic conditions (Larsen et al. 2011). There is also evidence to suggest that exercise efficiency is improved by increases in contractile efficiency (a lower ATP cost of force production) (Bailey et al. 2010), which could be related to alterations in calcium handling proteins. Our finding of a more prominent effect at lower exercise intensities is in agreement with near infra‐red spectroscopy studies where it was shown that dietary nitrate reduces the oxygen cost of low‐intensity exercise in humans (Bailey et al. 2010). In the current study, the observed changes mainly occurred in the soleus muscle which is predominantly composed of type 1 (slow twitch) muscle fibers, with a preference for oxidative metabolism as an energy source. In contrast, the gastrocnemius contains a relatively higher proportion of type 2 (fast twitch) fibers which tend to rely more on anaerobic energy sources (Edgerton et al. 1975; Scott et al. 2001). As noted, our results show effects of dietary nitrate on tissue oxygenation maximal change mainly in the more oxidative muscle at low exercise intensities. Such insight is not available from nonspatially resolved methods, such as near infra‐red spectroscopy.

In this study, physical activity was restricted to a confined range to avoid inter subject variability, as dietary nitrate has reduced effects on elite athletes (Peacock et al. 2012). To ensure correct assessment of the reproducibility with different levels of exercise intensities (Sanchez et al. 2009), this study derived the reproducibility from the same cohort group with identical experimental conditions. The quantitative fMRI method was chosen to isolate tissue oxygenation from other physiological parameters (Damon et al. 2007a, 2007b), with spatially resolved measurements in activated muscle groups. Physiological measurements were carried out immediately before each scan visit to determine the effects of systemic changes on observed measurements (Winter et al. 2011).

**Figure 4.**
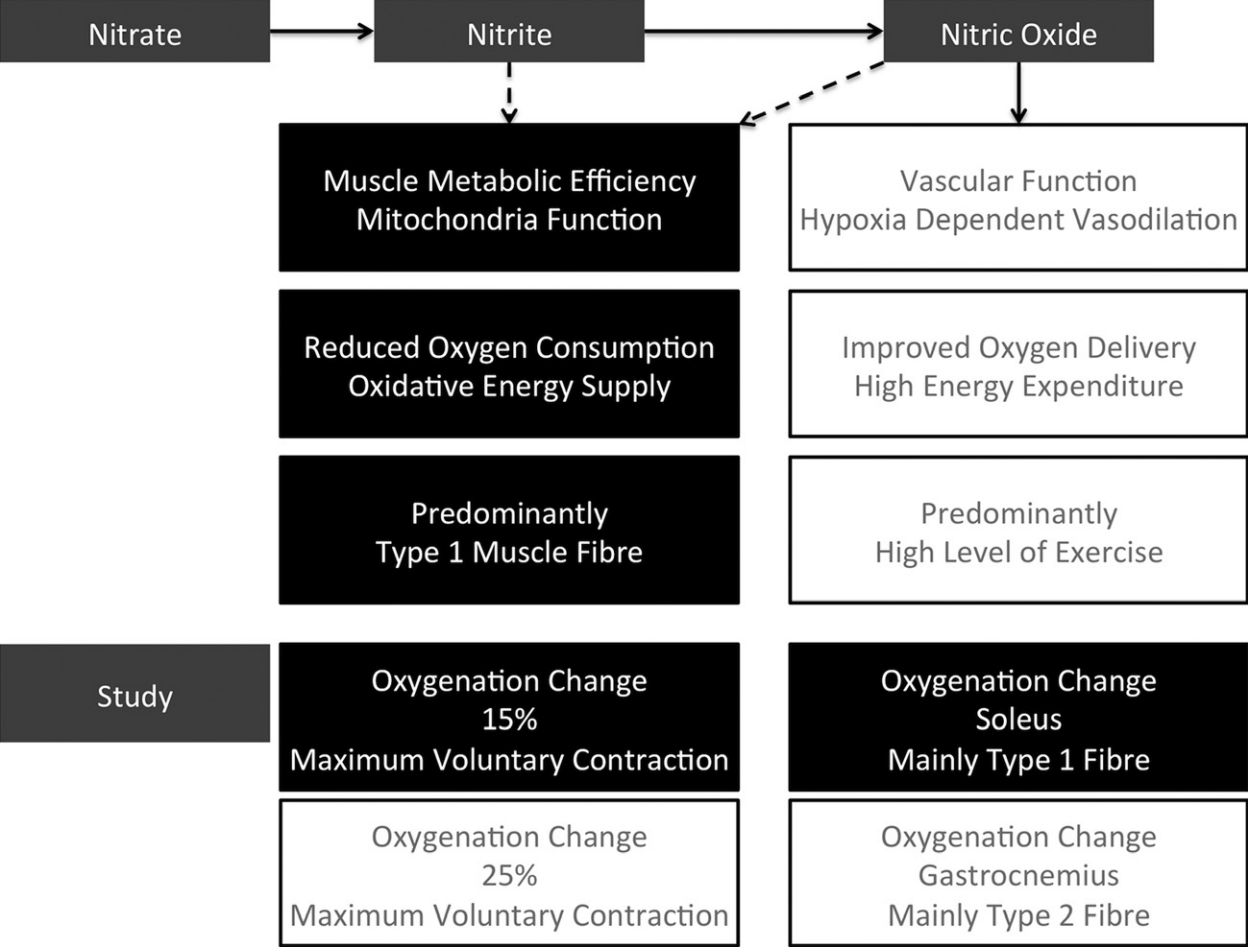
Schematic diagram of the study finding. Nitrate is first converted into nitrite and subsequently nitric oxide. Nitric oxide has known vascular effects, while the effects of nitrite and nitric oxide on mitochondria function is still under intensive research. Our finding that dietary nitrate reduces tissue oxygenation change primarily at lower exercise intensity in muscle groups predominantly composed of type 1 muscle fibers, indicates that hypoxia‐dependent vasodilation is minimal in healthy subjects. This supports the biomechanism that dietary nitrate improves muscle efficiency through mitochondria function enhancement.

This study, alongside previous data, indicates that energy efficiency during aerobic activity might be improved by dietary nitrate. Our data exclude a significantly enhanced exercise induced vasodilation effect in skeletal muscle from these healthy volunteers (Fig. 4). However, we cannot exclude the possibility that such an effect might be seen in patients with peripheral arterial disease, in whom microvascular oxygen tension is substantially lower, thereby favoring a greater reduction in nitrite to nitric oxide. Nevertheless, the oxygen sparing effect of nitrite per se may be of value in ischaemic situations.

Despite a relatively small number of subjects, the results revealed significant effects of dietary nitrate. However, future larger studies are required to assess the variations across wider populations. Furthermore, the two intensity levels employed in this study only provide a crude indication of the relationship between intensity and tissue oxygenation maximal change. Future studies incorporating a variety of stress levels may potentially discern the effects of dietary nitrate on aerobic/anaerobic activities. Moreover, it would be valuable to perform muscle biopsies and more invasive physiological measurements in the same experiment, so that imaging results can be directly compared with cellular chemistry and mitochondria function.

In this study, the intervention visit was designed to be 2.5 h after dietary nitrate consumption. While the plasma nitrite level was not measured it has been shown that plasma nitrite concentration peaks at 2–3 h after dietary supplementation (Webb et al. 2008), and similar protocols have been adopted in a number of studies (Webb et al. 2008; Engan et al. 2012; Wylie et al. 2013). However, the full dynamic effects of nitrite can be assessed through multiple time point measurements with plasma nitrite concentration measurement. The study design did not adopt a double blinded strategy, as indistinguishable placebo was not available on the open market. Furthermore, the nitrate supplementation was carried out as the last visit, given that the long‐term effects of dietary nitrate is unclear. It has been shown that significant residual drug effect is associated with agents that affecting skeletal muscle functions (Egner et al. 2013). To address this, the analysis protocol was predefined ahead of the study, and image analysis was carried out by a researcher independent from the experimental procedure and statistical analysis. Steady state of the muscle was achieved by use of the dummy cycle in the fMRI experiment. The MRI measure for the tissue oxygenation, the effective transverse relaxation rate, is also influenced by transverse relaxation time (Damon and Gore 2005). However, transverse relaxation time in skeletal muscle is known to increase with exercise intensity (Fisher et al. 1990). An hypoxia‐dependent vasodilation would increase transverse relaxation time and reduce transverse relaxation rate at higher exercise intensity, resulting in greater changes in the MRI measures. Therefore, the consideration of the change in transverse relaxation time, arising from water redistribution, would not alter the conclusion drawn. As the 15% MVC run was carried out first, there may have been a carry over into the 25% MVC trial, potentially blood flow.

In conclusion, dietary inorganic nitrate reduces the tissue oxygenation maximal change in skeletal muscle to physical exercise, with a more prominent effect at lower intensities and within muscle groups with predominantly type 1 fibers. This is consistent with a primary effect on mitochondrial efficiency rather than a vascular effect (Fig. 4). The effects observed in this study may also have arisen from a reduction in ATP cost of force production. The improved exercise efficiency may have clinical application in peripheral arterial disease and also in angina and heart failure if a similar increase in metabolic efficiency were to occur in heart muscle.

## Acknowledgment

The authors would like to thank Gordon Buchan for technical support, Baljit Jagpal, Nichola Crouch, Katrina Klaasen, and Beverley MacLennan for radiographic support.

## Conflict of Interest

None declared.
